# Magnetic resonance angiography with compressed sensing: An evaluation of moyamoya disease

**DOI:** 10.1371/journal.pone.0189493

**Published:** 2018-01-19

**Authors:** Takayuki Yamamoto, Tomohisa Okada, Yasutaka Fushimi, Akira Yamamoto, Koji Fujimoto, Sachi Okuchi, Hikaru Fukutomi, Jun C. Takahashi, Takeshi Funaki, Susumu Miyamoto, Aurélien F. Stalder, Yutaka Natsuaki, Peter Speier, Kaori Togashi

**Affiliations:** 1 Department of Diagnostic Imaging and Nuclear Medicine, Graduate School of Medicine, Kyoto University, Kyoto, Japan; 2 Department of Neurosurgery, National Cerebral and Cardiovascular Center, Suita, Japan; 3 Department of Neurosurgery, Graduate School of Medicine, Kyoto University, Kyoto, Japan; 4 Siemens ltd. China, Shanghai, China; 5 Siemens Medical Solutions USA, Inc., Huntington Beach, California, United States of America; 6 Siemens Healthcare, Erlangen, Germany; Universitatsklinikum Freiburg, GERMANY

## Abstract

Compressed sensing (CS) reconstructions of under-sampled measurements generate missing data based on assumptions of image sparsity. Non-contrast time-of-flight MR angiography (TOF-MRA) is a good candidate for CS based acceleration, as MRA images feature bright trees of sparse vessels over a well-suppressed anatomical background signal. A short scan time derived from CS is beneficial for patients of moyamoya disease (MMD) because of the frequency of MR scans. The purpose of this study was to investigate the reliability of TOF-MRA with CS in the evaluation of MMD. Twenty-two patients were examined using TOF-MRA with CS (CS-TOF) and parallel imaging (PI-TOF). The acceleration factors were 3 (CS3) and 5 (CS5) for CS-TOF, and 3 (PI3) for PI-TOF. Two neuroradiologists evaluated the MMD grading according to stenosis/occlusion scores using the modified Houkin’s system, and the visibility of moyamoya vessels (MMVs) using a 3-point scale. Concordance was calculated with Cohen’s *κ*. The numbers of MMVs in the basal ganglia were compared using Bland-Altman analysis and Wilcoxon’s signed-rank tests. MRA scan times were 4:07, 3:53, and 2:42 for PI3, CS3, and CS5, respectively. CS-reconstruction completed within 10 minutes. MMD grading and MMV visibility scales showed excellent correlation (*κ* > .966). Although the number of MMVs was significantly higher in CS3 than in PI3 (*p* < .0001) and CS5 (*p* < .0001), Bland-Altman analysis showed a good agreement between PI3, CS3, and CS5. Compressed sensing can accelerate TOF-MRA with improved visualization of small collaterals in equivalent time (CS3) or equivalent results in a shorter scan time (CS5).

## Introduction

Compressed sensing (CS) has been vigorously studied in recent years because of its potential to reduce scan time[1] or to improve spatio-temporal resolution while maintaining the same scan time[2]. The goal of CS is to approximate the image quality of a fully sampled measurement from under-sampled *k*-space data, by exploiting the intrinsic sparsity in the imaged structures in a non-linear iterative regularized reconstruction. Several studies[3,4] on contrast enhanced MRA with CS have reported on its excellent speed (e.g. only 10 seconds were needed to cover the whole brain[5]). Non-contrast time-of-flight (TOF) MRA is also a good candidate for CS based acceleration, as images feature a bright trees of sparse vessels over a well suppressed anatomical background signal[6–10]. In a preceding study on normal volunteers[11], the scan time of cerebrovascular non-contrast TOF-MRA with CS (CS-TOF) was reduced to approximately half of that of TOF-MRA with parallel imaging (PI-TOF), the current clinical standard for accelerating acquisition[12–14], while maintaining the image quality.

CS reconstructions of accelerated (i.e., under-sampled) measurements generate the missing data by making assumptions about the image sparsity after a certain transformation; in our case, magnitude images were sparsified in the wavelet domain of the magnitude image. Therefore, the diagnostic capability of CS-TOF requires validation, especially in regard to whether or not it introduces an over- or under-estimation of vascular pathological changes in comparison with clinically established methods. For cerebrovascular aneurysms, images acquired with CS-TOF have been successfully evaluated[15,16]. Our aim here is to extend this validation to vascular stenosis and small vessels.

Moyamoya disease (MMD) is an uncommon cerebrovascular disorder characterized by progressive stenosis and occlusion of the supra-clinoid internal carotid artery and its main branches within the circle of Willis. These changes result in the formation of a fine vascular network, known as the moyamoya vessels (MMVs), and other collateral circulations such as superficial temporal artery. Visualization of these is directly linked to the surgical treatments[17]. Although digital subtraction angiography (DSA) still remains the gold standard for the diagnosis and staging of MMD, TOF-MRA is now considered to be a reliable alternative with high sensitivity and specificity[18–20]. TOF-MRA is a non-invasive modality that can be readily applied to asymptomatic MMD patients and repeatedly used for follow-up examinations, even in children.

Therefore, we hypothesized that CS-TOF can be used to assess MMD, and examined the grading and visualization of MMVs in a prospective MMD cohort, comparing the results from CS-TOF with those from conventional PI-TOF, with the CS-TOF using the same or a higher acquisition acceleration factor than the PI-TOF.

## Materials and methods

### Study population

This study was approved by the local institutional review board (Kyoto University Graduate School Ethics committee), and written informed consent was obtained from all subjects. From December 2014 through to October 2015, 22 patients with MMD were enrolled (mean age, 43.1 years; age range, 29–65 years; 14 women and 8 men). All subjects were scanned using both CS-TOF and PI-TOF in the same session. Diagnosis of MMD was confirmed by DSA or conventional MRA (PI-TOF). Ten of the patients had undergone a previous operation for direct bypass from the superficial temporal artery to the middle cerebral artery. Exclusion criteria were failure to acquire the whole imaging sets, as well as large motion and susceptibility artifacts.

### Acquisition of MR angiography

**Fig 1:**
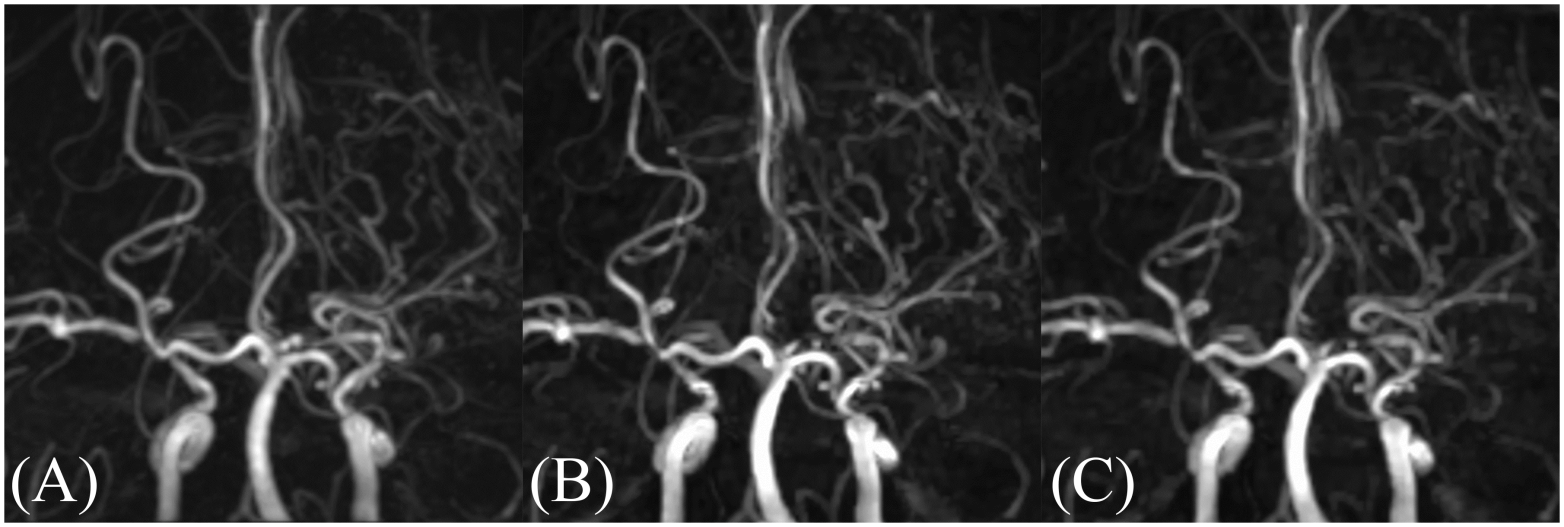
Coronal views of MIP images. (A) PI3, (B) CS3, (C) CS5. Occlusion of the left MCA and the development of MMVs are equivalently visualized in these images. PI3, conventional TOF MRA with an acceleration factor of 3; CS3, compressed sensing TOF MRA with an acceleration factor of 3; CS5, compressed sensing TOF MRA with an acceleration factor of 5; MCA, middle cerebral artery.

CS-TOF and PI-TOF were acquired using a 3T MR scanner (MAGNETOM Skyra, Siemens Healthcare, Erlangen, Germany) with a 32-channel head coil (Fig 1). A research sequence and reconstruction prototype provided by Siemens Healthcare GmbH (Erlangen, Germany) was used for CS-TOF. Common acquisition parameters were as follows: TR/TE = 20/3.69 ms, FA = 18°, 4 slabs, TONE ramp, 70%; and a reconstructed voxel size of 0.38 × 0.38 × 0.38 mm. For CS-TOF, the FOV was 220 × 200 × 63 mm and the acquired matrix was 288 × 264 × 82 (interpolated to 576 × 528 × 164), whereas for PI-TOF, the FOV was 240 × 200 × 62 mm, and the matrix was 320 × 270 × 81 (interpolated to 640 × 540 × 162).

**Fig 2:**
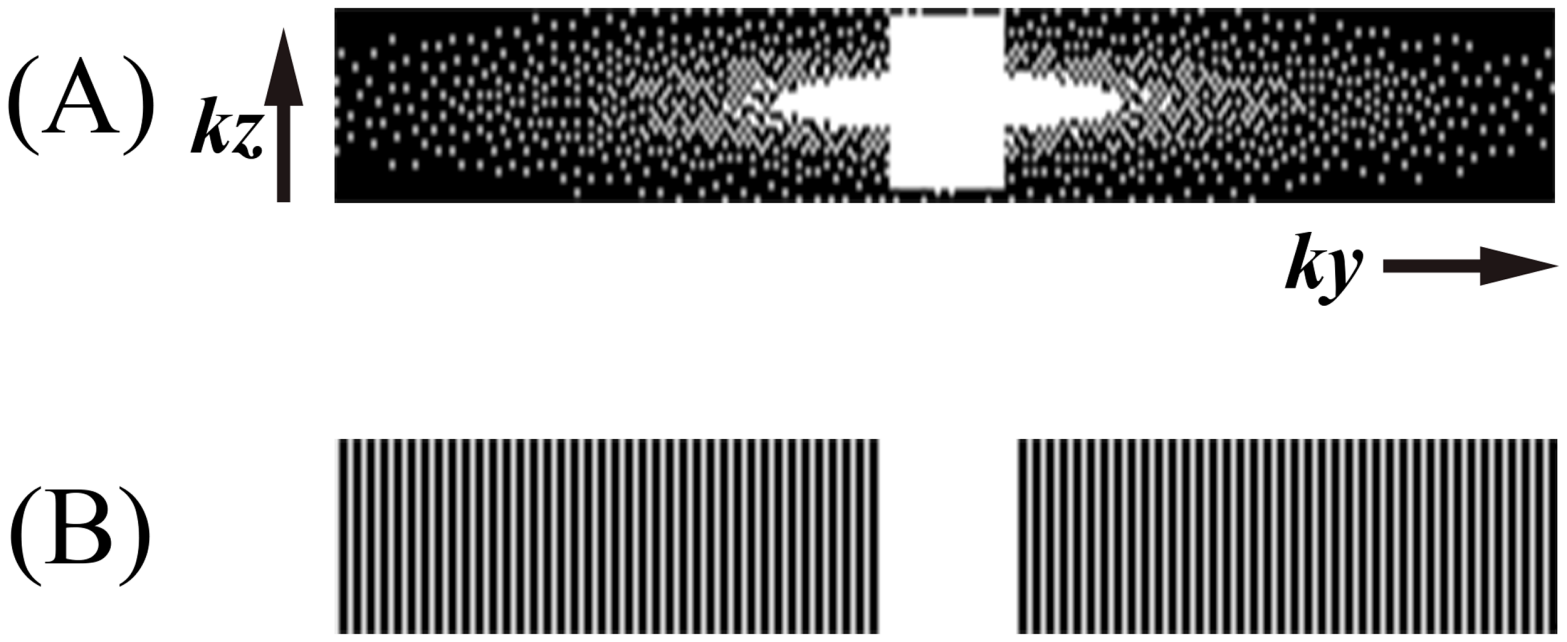
Sampling patterns of the slab. *Ky-Kz* plane of k-space. (A) Compressed Sensing, (B) Parallel Imaging.

For CS-TOF data acquisition, a conventional 3D TOF gradient-echo sequence was extended to facilitate incoherent sampling of *k*-space using a variable-density Poisson disk sampling pattern in the *ky-kz* encoding plane on a Cartesian grid[21] (Fig 2). After data acquisition, the image was reconstructed from the under-sampled data by nonlinear iterative SENSE-type reconstruction[14]. The reconstruction was implemented inline on the scanner. The following minimization problem is solved using a modified Fast Iterative Shrinkage-Thresholding Algorithm (mFISTA) [22]:

$$\underset{x}{\text{min}}\;{\left|\sum_{j}^{N}y_{j}-F_{u}S_{j}x\right|}_{2}^{2}+\lambda {\left|Wx\right|}_{1}$$

where *x* is the image to reconstruct, *y*_*j*_ and *S*_*j*_ are the *k*-space data and coil sensitivity for *j*-th coil element, *Fu* is the Fourier under-sampling operator, *W* is the redundant Haar wavelet transform, and *λ* is the normalized regularization parameter (set to 0.008). This equation and the parameter was the same and optimized in our previous study[11]. The image reconstruction was fully integrated with the scanner software and was performed inline.

The acceleration rates for CS-TOF were 3 (CS3) and 5 (CS5), while that of PI-TOF was 3 (PI3). PI3 (acceleration in phase encoding direction only) was selected as the reference standard, as it is the acceleration factor in general use in our institution. PI-MRA was reconstructed with the scanner’s product implementation of GRAPPA (Generalized Auto-Calibrating Partially Parallel Acquisition) [13,23].

### Image analysis

Firstly, two neuroradiologists (T.O. and Y.F., with 26 and 19 years of experience, respectively) graded the stenotic changes at Circle of Willis on both CS-TOF and PI-TOF. MMD grading was assessed according to the modified Houkin’s grading system[24]. This classifies the progress of the disease into 4 grades based on the total stenosis/occlusion scores of internal carotid artery, middle cerebral artery, anterior cerebral artery, and posterior cerebral artery.

**Fig 3:**
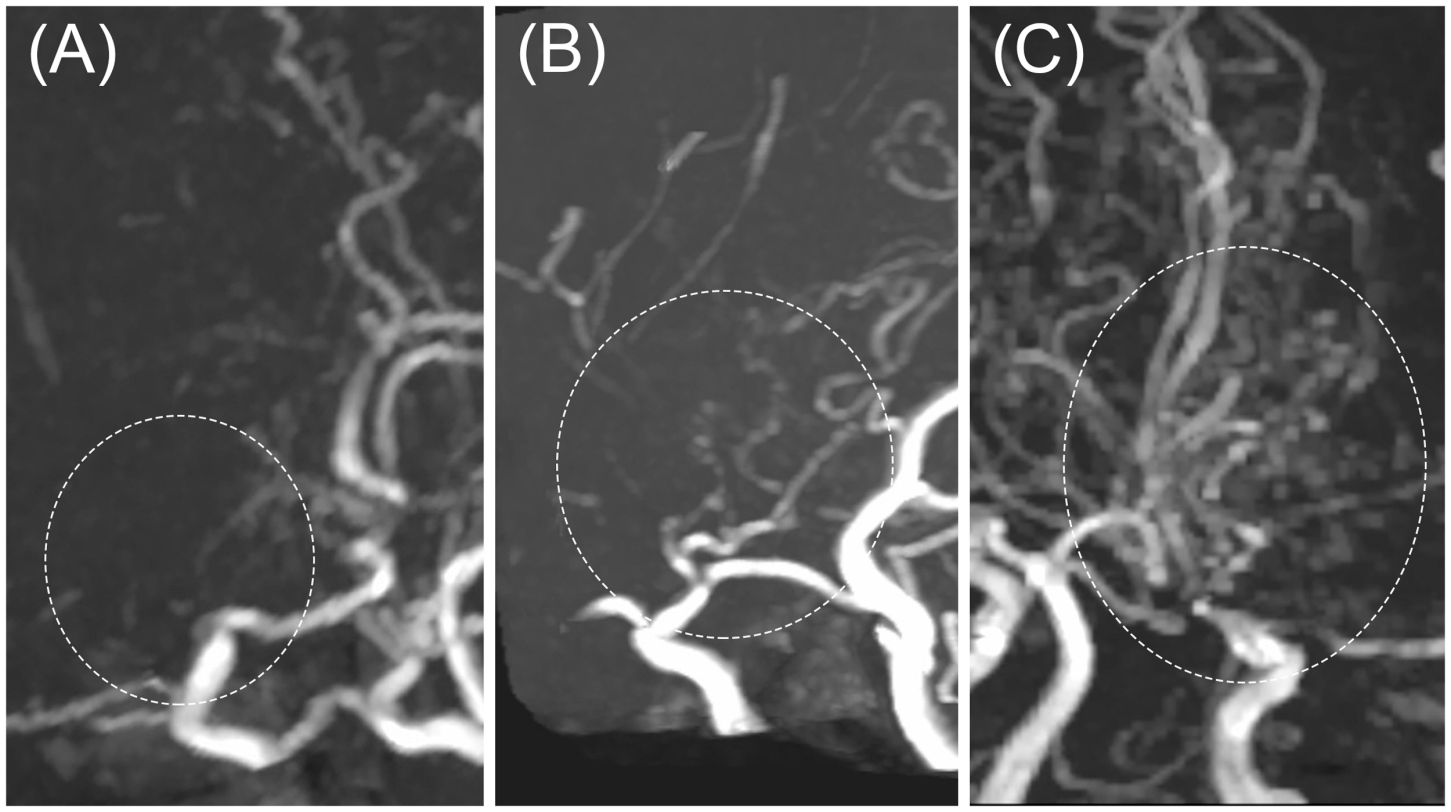
Moyamoya vessels (MMVs) on MIP images. (A) Few or no MMVs, (B) a moderate amount of MMVs (weak vessels visibility, but sufficient for diagnosis), (C) a large amount of MMVs (vessels are clearly visualized). All images are compressed sensing TOF-MRA with an acceleration factor of 3.

Secondly, MMV visibility was scored using a 3-point scale: 1, few or no MMVs; 2, a moderate amount of MMVs (weak visualization of vessels, but sufficient for diagnosis); 3, a large amount of MMVs (vessels clearly visualized). Examples are presented in Fig 3.

Thirdly, high-intensity spots recognized as vessels (perforating arteries or MMVs in the basal ganglia) were manually counted on an original 2D slice at the level of the basal ganglia by two neuroradiologists (T.Y. and S.O., with 11 and 11 years’ experience, respectively). For each patient, this was performed separately for the left and right side, with the vessels being tracked through upper and lower slices to differentiate vessels from noises. Definite vessels were counted as 1, and probable vessels as 0.5.

Finally, the visibility of bypass vessels was also subjectively evaluated in the 10 patients who had undergone previous bypass surgery.

All of the analysis above was performed independently, and the sequence was blinded for the raters.

### Statistical analysis

The Cohen’s *κ* statistic (quadratic weighted) was used to analyzed for the MMD grading and for the MMV visibility on maximum intensity projection (MIP) images, facilitating comparisons between CS3, CS5, and PI3 (slight agreement, κ < 0.2; fair agreement, 0.2–0.4; moderate agreement, 0.4–0.6; substantial agreement, 0.6–0.8; almost-perfect agreement, ≥0.8). The MMV numbers of CS3 and CS5 were compared with PI3 using the Bland-Altman analysis and the Wilcoxon signed rank test. Inter-rater agreement in the MMV numbers was also evaluated by intraclass correlation coefficient (ICC). Statistical analyses were performed using R (version 3.3.1, https://www.r-project.org/), with a *p* value < .05 being considered as statistically significant.

## Results

MRA scan times were 4:07, 3:53 and 2:42 for the PI3, CS3 and CS5 acquisitions, respectively (Fig 2). The CS-TOF reconstruction was started at the end of the acquisition and finished within 10 minutes. No patients were excluded from the analysis.

The modified Houkin’s MMD grading of CS3 and CS5 showed a nearly complete agreement with those of PI3 (*κ* = .993 and .996, respectively; see Table 1), thereby demonstrating equivalent grading capabilities. Inter-rater agreements were substantial (Table 2).

**Table 1:** The results of the MRA grading of moyamoya disease with PI3, CS3, and CS5.

	PI3			
CS3 (CS5)	Stage 1	Stage 2	Stage 3	Stage 4
Stage 1	8 (8)			
Stage 2		26 (24)		
Stage 3		0 (2)	32 (31)	
Stage 4			1 (2)	21 (21)

Two observers independently assessed 44 sides of 22 patients, thus a total of 88 results are illustrated. The κ value was 0.993 between PI3 and CS3, and 0.966 between PI3 and CS5. PI3, conventional TOF MRA with an acceleration factor of 3; CS3, compressed sensing TOF MRA with an acceleration factor of 3; CS5, compressed sensing TOF MRA with an acceleration factor of 5.

**Table 2:** Inter-rater agreement in the grading score of moyamoya disease observed as Cohen’s κ statistic.

		Rater 2		
		PI3	CS3	CS5
Rater 1	PI3	.755	.751	.765
	CS3	.748	.748	.758
	CS5	.745	.741	.757

Substantial agreement was shown for each combination of PI3, CS3, and CS5.

PI3, conventional TOF MRA with an acceleration factor of 3; CS3, compressed sensing TOF MRA with an acceleration factor of 3; CS5, compressed sensing TOF MRA with an acceleration factor of 5.

MMV visibility on CS-TOF MIP images was also equivalent to that on PI-TOF images (Table 3). Excellent agreements were obtained between PI3 and CS3 (κ = .992), and between PI3 and CS5 (κ = .967). Inter-rater agreements were substantial (Table 4).

**Table 3:** The visibility of moyamoya vessels on MIP images from PI3, CS3, and CS5.

	PI3		
CS3 (CS5)	Grade 1	Grade 2	Grade 3
Grade 1	33 (33)	1 (1)	
Grade 2	0 (2)	27 (25)	0 (1)
Grade 3			27 (26)

The κ value was 0.992 between PI3 and CS3, and 0.967 between PI3 and CS5.

PI3, conventional TOF MRA with an acceleration factor of 3; CS3, compressed sensing TOF MRA with an acceleration factor of 3; CS5, compressed sensing TOF MRA with an acceleration factor of 5.

**Table 4:** Inter-rater agreement on the visibility of moyamoya vessels observed as Cohen’s κ statistic.

		Rater 2		
		PI3	CS3	CS5
Rater 1	PI3	.608	.608	.608
	CS3	.631	.631	.631
	CS5	.608	.608	.608

Substantial agreement was shown for each combination of PI3, CS3, and CS5.

PI3, conventional TOF MRA with acceleration factor of 3; CS3, compressed sensing TOF MRA with acceleration factor of 3; CS5, compressed sensing TOF MRA with acceleration factor of 5.

**Fig 4:**
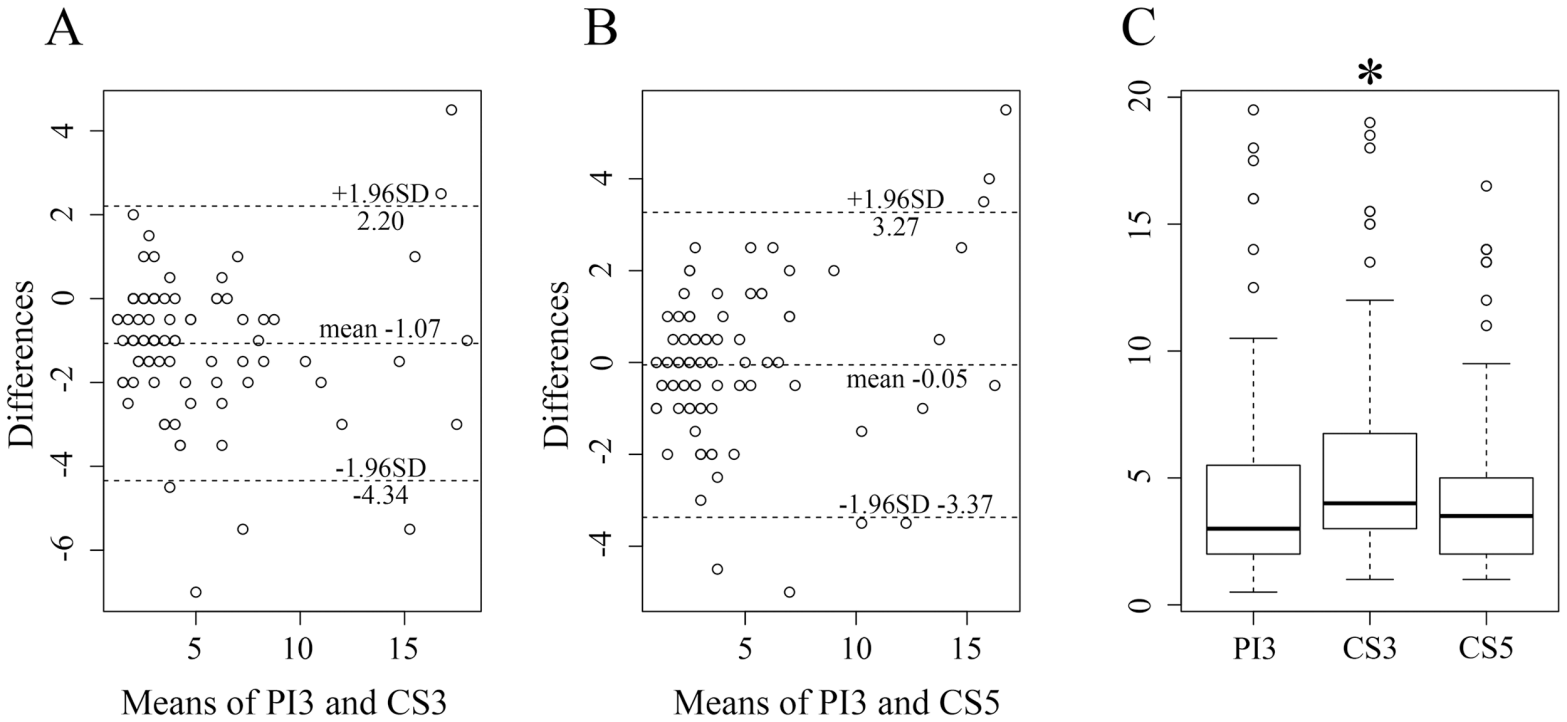
Bland-Altman plots and Box-and-whisker plot of the numbers of high-intensities, which are considered as vessels, observed in the basal ganglia. (A, B) Bland-Altman plots. The Y-axis unit (differences) is numerical. (A) No significant bias was found comparing PI3 with CS3. (B) Hardly any differences were detected when comparing PI3 with CS5. (C) Box-and-whisker plot. The number for CS3 was significantly higher than that of PI3 (*p* < .0001) and CS5 (*p* < .0001). PI3, conventional TOF MRA with an acceleration factor of 3; CS3, compressed sensing TOF MRA with an acceleration factor of 3; CS5, compressed sensing TOF MRA with an acceleration factor of 5.

The median number of MMVs at the basal ganglia level was 3 for PI3 (1st–3rd quartile: 2.0–5.3), 4 for CS3 (3.0–6.6), and 3.5 for CS5 (2.0–5.0). The Bland-Altman analysis for the number of MMVs also showed a good agreement. The number of MMVs on CS3 was significantly higher than that on PI3 (*p* < .0001) or that for CS5 (*p* < .0001) (Fig 4). No significant difference was observed between CS5 and PI3 for the number of MMVs. The inter-rater ICC for counting of the number of MMVs showed an excellent correlation (.894).

All of the bypass vessels of the patients who had undergone operations were well recognized in all three acquisitions. The superficial temporal arteries were also satisfyingly depicted in the other patients.

## Discussion

We investigated the diagnostic quality of CS-TOF by evaluating MMD grading (which include stenosis/occlusion scores) and MMV visibility on both MIP images and 2D slices at the level of the basal ganglia. CS-TOF better visualized MMVs that work as small collaterals in an equivalent scan time, and attained equivalent visualization in reduced scan time of less than 3 minutes.

The choice of an acceleration rate of 3 or 5 for CS-TOF had little effect on the visual aspect and the grading of MMD, suggesting the absence of either over- or under-estimation due to irregular undersampling and nonlinear iterative reconstruction. In 4 cases (9.1%), MMD grading based on CS5 resulted in an over-diagnosis (2 cases, stage 2 as 3; 2 cases, stage 3 as 4; see Table 1), but we did not observe any cases of under-diagnosis. Thus, if CS-TOF shows a severe stenosis, a confirmation of the finding using contrast-enhanced angiography (MR, CT, or DSA) would be worth considering for the initial evaluation, but is not required for follow-up observations focusing on relative changes. The inter-rater correlation for the stenosis/occlusion scores was substantial; however, the intra-rater agreement was almost perfect. Therefore, the difference in scoring is considered to reflect variations in evaluation due to the individual interpreting the images, and this may have contributed to the over diagnoses in the CS5 cases.

The presence and quantity of MMVs is also important for the evaluation of MMD[25–27], although it is not easy to evaluate it using TOF-MRA[28]. The visibility of MMVs on MIP images was almost the same on PI-TOF and CS-TOF, whereas the number of MMVs counted on the CS3 images was significantly higher than that on the PI3 images. This evaluation had high intra-observer agreement, with inter-observer reproducibility showing substantial agreement, probably because evaluation on the MIP images is tended to be influenced by observer subjectivity, as in the evaluation of stenosis/occlusion. The evaluation of the number of MMVs in basal ganglia helps to understand the difference in characteristics between CS-TOF and PI-TOF. CS3 showed more MMVs than the others; this is likely to be due to the inherent denoising effect of CS[1]. CS reconstruction enhances the contrast of vessels by counteracting background noise, while this denoising effect may also simultaneously diminish signal from vessels. The previous study reported that the apparent SNR of CS-MRA is higher than that of PI-MRA with more than 3-fold acceleration[11]. The high apparent SNR is considered to result in more moyamoya vessels being visualized in CS. In CS3, the positive effect was considered to exceed the negative effect, while even in CS5, the numbers of MMVs were comparable with those on PI3. A previous report supports this argument, as it showed that attenuation of small vessel signals is strong on CS-TOF in comparison with PI-TOF[11].

**Fig 5:**
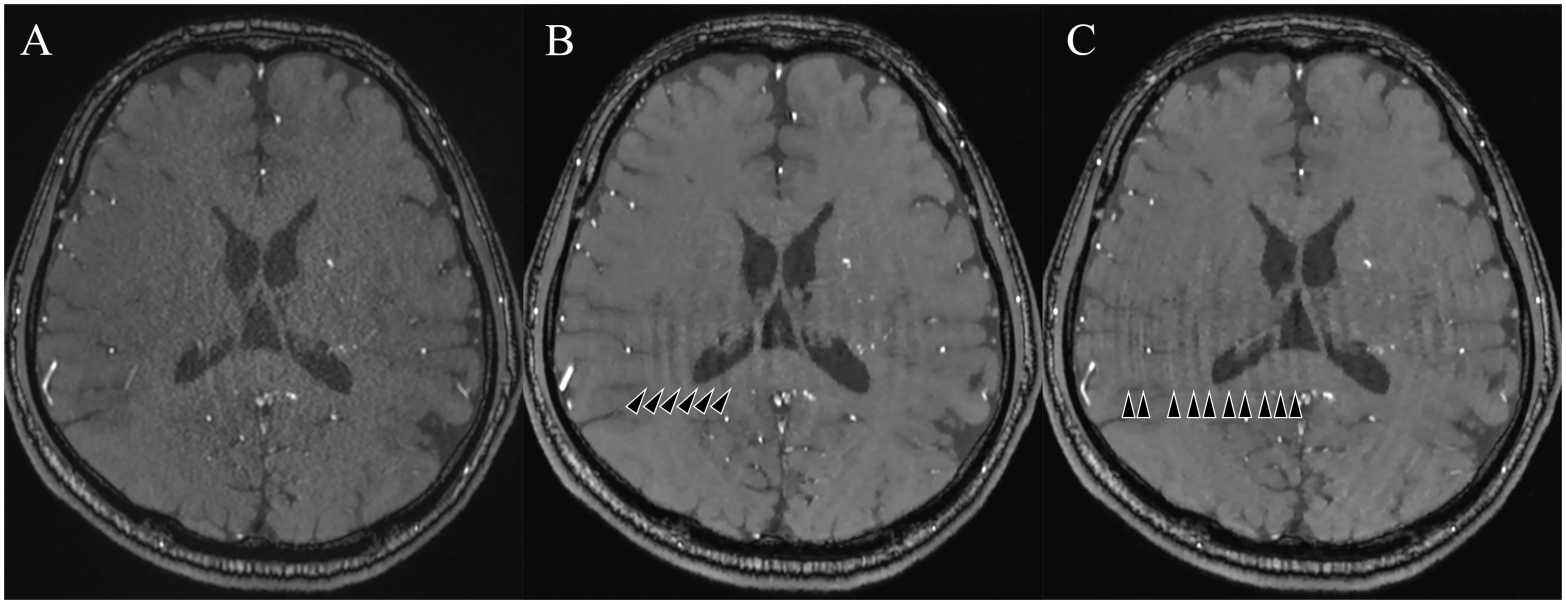
The 2D slice images at the level of the basal ganglia for (A) PI3, (B) CS3, and (C) CS5. On CS images, some artifacts such as curved stripe patterns (arrowheads) were seen. These artifacts were not observable on the MIP images. PI3, conventional TOF MRA with an acceleration factor of 3; CS3, compressed sensing TOF MRA with an acceleration factor of 3; CS5, compressed sensing TOF MRA with an acceleration factor of 5.

**Fig 6:**
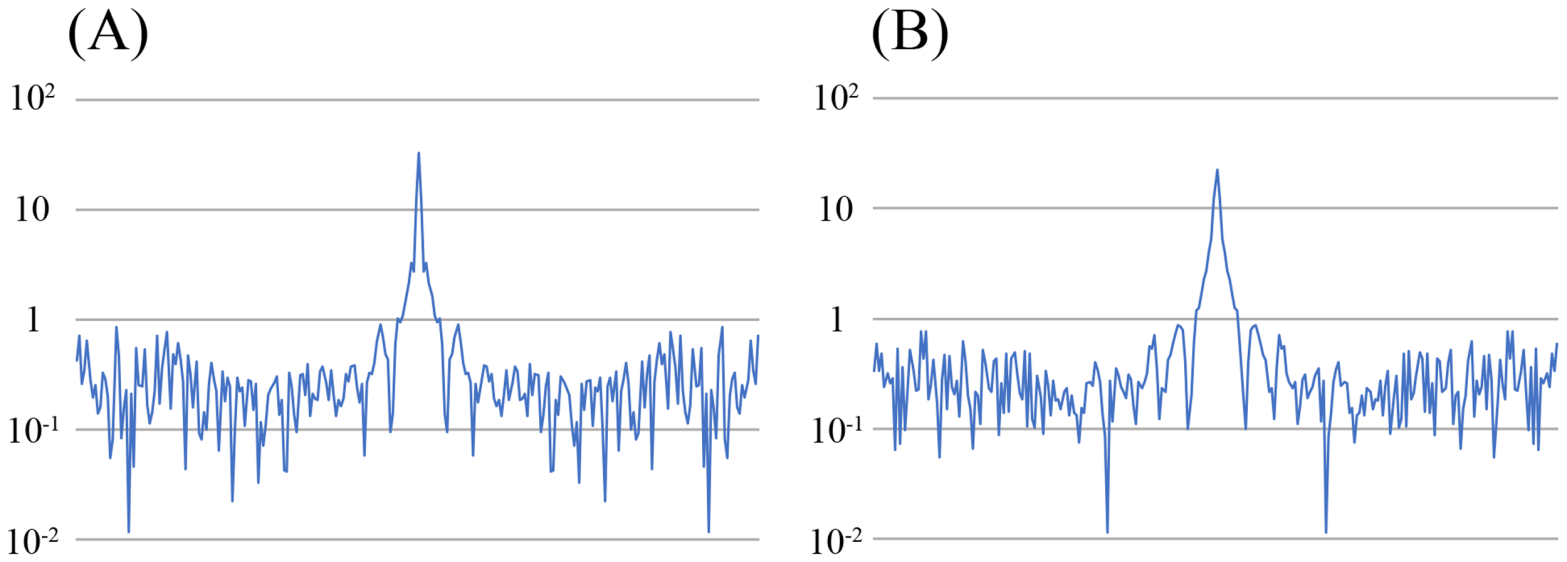
Point spread functions of the sampling pattern. (A) CS3, (B) CS5. The ratio of the main peak to the standard deviation of the pseudo-noise (incoherent artifacts) is 74.2 for CS3, and 35.1 for CS5. CS3, compressed sensing TOF MRA with an acceleration factor of 3; CS5, compressed sensing TOF MRA with an acceleration factor of 5.

Fig 5A shows representative images for all three acquisitions. The example PI3 image in Fig 4A shows prominent speckled noise, especially in the center[14,29]; this is due to the g-factor increase in the center. The example CS images of Fig 5B and 5C show highly reduced noises throughout the image; however, curved stripe patterns consistent with ghosts originating from the skull boundaries are apparent, with these becoming most prominent at the center. The intensity of this artifact varied from slice to slice, but the direction of the stripe patterns always aligned with the phase encoding direction. We thus speculate that these artifacts are residual aliasing artifacts originating from the sparse undersampling, possibly derived from the outer edge of the skull. We evaluated the incoherence by the point spread function of sampling patterns of CS (Fig 6), and the ratio of the main peak of point spread function to the standard deviation of the pseudo-noise (incoherent artifacts) is 74.2 for CS3, and 35.1 for CS5. The limited slab size (28 slices, 16.7% oversampling) might constraint the incoherence. Slight patient movements are also a conceivable reason. Increasing the regularization parameters used in the reconstruction can mitigate this artifact, but a strong increase may obscure small vessels. However, this artifact is hardly noticeable on the MIP images (Fig 1), and did not affect the detection of the vessels in the basal ganglia in this study.

The total reconstruction time for CS was approximately 10 minutes, which is too long for clinical use. CS-TOF is scanned in 4 slabs in this study, and slab-wise reconstruction after the acquisition of each slab cloud reduce the actual waiting time. Moreover, the reconstruction time could be drastically shortened to clinically acceptable times by using a graphics processing unit implementation, which was not available in this study.

There are several limitations to this study. Firstly, CS and PI were separately evaluated for comparison in this study, however, CS and PI are not essentially exclusive technique, but they can be used synergistically for further acceleration[30]. Secondly, the acquisition matrix was not the same between CS-MRA and PI-MRA (voxel size was adjusted to be the same). Setting the same values for both acquisition matrix and spatial resolution was not feasible due to the limitation of the sequences at the moment we collected the data. Thirdly, not all patients underwent DSA. Only 8 subjects had DSA within 6 months before and after examination of CS-MRA. DSA is invasive, and in the situation where MRI/MRA can depict time-course changes and offer reliable MMD grading, it is not considered essential. Furthermore, several studies[31,32] have verified a good correlation between MRA (Houkin’s criteria) and DSA (Suzuki’s criteria[33]). Fourthly, a relatively small number of patients were assessed, but it is considered sufficient to elucidate the capability of CS-TOF. Fifthly, only two CS-TOF acceleration rates (3 and 5) were evaluated. The research scans were added to the routine scan, and further additions were considered ethically inappropriate because of the burden on the patients.

In conclusion, compressed sensing can accelerate TOF-MRA, while preserving the diagnostic capability for MMD. CS-TOF better visualized collateral MMVs in the equivalent acceleration. Moreover, five-fold accelerated CS-TOF was considered as acceptable for the proper diagnosis of MMD, allowing detection of steno-occlusive changes and MMVs. Shortening the scan time without reducing the clinically required coverage and spatial resolution brings benefits to patients, especially those who need to receive repeated life-time follow-up examinations. Further research is needed to investigate whether even higher acceleration rates can be used reliably in clinical practice.
